# Complete Genome Sequence of *Enterococcus thailandicus* Strain a523 Isolated from Urban Raw Sewage

**DOI:** 10.1128/genomeA.01298-17

**Published:** 2017-11-22

**Authors:** Gustavo Ybazeta, Laura Douglas, Jenna Graham, Nya L. Fraleigh, Yanal Murad, Justo Perez, Francisco Diaz-Mitoma, Kim Tilbe, Reza Nokhbeh

**Affiliations:** a Health Sciences North Research Institute, Sudbury, Ontario, Canada; b Health Sciences North Hospital, Sudbury, Ontario, Canada; c Northern Ontario School of Medicine, Sudbury, Ontario, Canada; d CREM Co. Laboratories, Mississauga, Ontario, Canada; e VBI Vaccines Inc., Cambridge, Massachusetts, USA

## Abstract

We report here the first complete circularly closed genome sequence of *Enterococcus thailandicus* strain a523 isolated from raw urban sewage. This genome contains 2,646,250 bp with a G+C content of 36.8%, 2,499 genes, 2,370 protein-coding sequences, 6 rRNA operons, 65 tRNAs, and 6 clustered regularly interspaced short palindromic repeat arrays.

## GENOME ANNOUNCEMENT

We present here the first full genome sequence of *Enterococcus thailandicus* isolated from urban raw sewage. Comparison of this genome sequence to previously reported draft genomes of isolates from cattle (1) and fermented sausages showed differences (GenBank accession no. NZ_JXLE00000000). Indexed paired-end shotgun DNA libraries (2 × 125 bp) were prepared using the NEB Ultra II kit (New England Biolabs, Inc.) and sequenced using the Illumina HiSeq 2500 paired-end 125-bp platform. In addition, the Oxford Nanopore MinION sequencing platform (2D sequencing) was used to generate long sequence reads (2). The Unicycler version 0.4.0 (3) and SPAdes version 3.10.1 (4) pipelines were used to assemble the genome. A hybrid alignment strategy was employed to assemble the genome using long reads generated by MinION, which served as scaffolds to accurately determine the order of Illumina HiSeq contigs and to close the genome. A total of 15,845,454 HiSeq reads (125 bp, paired end) and 15,163 MinION long reads were used in the *de novo* assembly of the *E. thailandicus* strain a523 genome with 798-fold average coverage per base pair. The closed genome contains 2,646,250 bp with a G+C content of 36.8%. Genome-wide open reading frames (ORFs) were predicted and annotated using Prokka version 1.12 (5) and the NCBI Prokaryotic Genome Automatic Annotation Pipelines (PGAAP), leading to the identification of 2,499 genes, 2,370 coding sequences, and 42 pseudogenes.

The genome of *E. thailandicus* a523 presents 6 rRNA operons, 65 tRNAs, and 1 transfer-messenger RNA. We identified several clustered regularly interspaced short palindromic repeat (CRISPR)-associated (Cas) systems, which included type II CRISPR-*cas9*, type II-*cas1*, *cas2*, and type II A-*Csn2*. Six arrays of repeats were detected that contained 16, 9, 8, 5, 7, and 6 repeats per array, respectively. The search for prophages, using Phaster (6, 7), identified a chromosomal island corresponding to a prophage sequence of 19.7 kb with a total of nine ORFs, of which six were annotated. We also identified at least four genomic cassettes associated with the production of bacteriocins. Searching within the Virulence Factor Database (VFDB) (8), analysis of this genome appeared to encode a hemolysin-like protein. Alignment of the amino acid sequence of this protein against the NCBI nonredundant protein database produced 99% identity to multispecies hemolysin III from *Bacillus cereus*. This alignment was further verified in Seaview (9). The Comprehensive Antibiotic Resistance Database (10, 11) was used to identify genes potentially implicated in antimicrobial resistance. This search produced three hits requiring *in vitro* validation for their activities. These include an aminoglycoside phosphotransferase (APH) and nucleotidyltransferase (ANT), a tetracycline target protection protein, and the lincosamide nucleotidyltransferase Lnu(G).

Given the adaptive versatility of enterococci in diverse niches, and the scarcity of *E. thailandicus*-related publications, releasing this first completed genome will assist researchers in better understanding the biology of *E. thailandicus* and its role in multibacterial communities.

### Accession number(s).

The complete genome sequence for *E. thailandicus* strain a523 was deposited in GenBank under accession number CP023074. 
